# Evaluation of patients undergoing abdominal sacrocolpopexy due to symptomatic pelvic organ prolapse in terms of gastrointestinal complications

**DOI:** 10.12669/pjms.344.15489

**Published:** 2018

**Authors:** Abdurrahman Hamdi Inan, Emrah Beyan, Adnan Budak, Ahkam Goksel Kanmaz

**Affiliations:** 1 Abdurrahman Hamdi Inan, Medical Doctor, Department of Obstetrics and Gynecology, Tepecik Training and Research Hospital, Izmir, Turkey; 2 Emrah Beyan, Medical Doctor, Department of Obstetrics and Gynecology, Tepecik Training and Research Hospital, Izmir, Turkey; 3 Adnan Budak, Medical Doctor, Izmir Provincial Health Directorate, Izmir, Turkey; 4 Ahkam Göksel Kanmaz, Medical Doctor, Department of Obstetrics and Gynecology, Tepecik Training and Research Hospital, Izmir, Turkey

**Keywords:** Sacrocolpopexy, Gastrointestinal complications, Intestinal injury, Postoperative nausea and vomiting

## Abstract

**Objectives:**

To evaluate the intra- and postoperative gastrointestinal complications following abdominal sacrocolpopexy and determine the possible causes.

**Methods:**

A total of 86 patients who underwent abdominal sacrocolpopexy due to symptomatic pelvic organ prolapse between January 2014 and January 2016 at İzmir Tepecik Training and Research Hospital Obstetrics and Gynecology Clinic were retrospectively reviewed using the hospital information system. Patients were divided into two groups: those with and without prolonged length of hospital stay. They were evaluated in terms of gastrointestinal complications and risk factors.

**Results:**

The reason for prolonged hospitalization was nausea and vomiting in 24 (88%) of 27 patients. The symptoms in these patients were recovered with hydration, stopping of oral intake, and administration of antiemetics. Nasogastric decompression and parenteral nutrition were required in three (11%) patients due to clinical and radiological evidence of ileus. The parameter that significantly prolonged the length of hospital stay was prior abdominal surgery (p < 0.05).

**Conclusion:**

There were obvious gastrointestinal complications in three out of 27 patients with prolonged length of hospital stay. These findings may be beneficial for preoperative patient counselling.

## INTRODUCTION

Pelvic organ prolapse (POP), defined as the downward herniation of female pelvic organs, is a condition that seriously affects the quality of life of females; its true prevalence is unknown. However, symptomatic POP prevalence is estimated to be 2.9%.^1^ The most common risk factors for the development of POP include parity, advanced age, and obesity.^2^

Vaginal delivery is the most frequently related risk factor for POP, wherein each vaginal delivery confers a 1.2-fold increase in the risk of developing prolapse.^2^ However, despite the association between vaginal delivery and POP, symptomatic prolapses can occur long after delivery, and there is no prolapse in most females who undergo vaginal birth.^3^ Aging, hypoestrogenism, and degenerative and organic diseases, whose prevalence increases with aging, increase the risk of developing POP.^4^ The frequency of surgery for prolapse is 0.1% in the age group of 20–29 years, whereas this rate is as high as 11.1% in the age group of 70–79 years.^5^

There is no consensus on whether hysterectomy causes POP. However, there are studies suggesting an increased risk of vaginal cuff prolapse in subjects who previously underwent hysterectomy.^6^

It is known that abdominal sacrocolpopexy, which is the gold standard for apical prolapse, can have major operative complications.^7^,^8^ Postoperative gastrointestinal complications may include nausea, inability to tolerate oral intake, vomiting, abdominal distension and discomfort, ileus, and small bowel obstruction (SBO).^9^ Radiologically, differentiation of ileus from SBO is very difficult; their symptoms can mimic each other, and both the conditions are generally treated conservatively.^10^ These complications increase the length of hospital stay and decrease patient comfort.^11^ Postoperative ileus may be caused due to many reasons, including surgical trauma (sympathetic hyperactivity), systemic endocrine response, inflammatory cytokines, general anesthesia, and opioid drug use.^12^ The objective of our study was to evaluate the gastrointestinal complications following abdominal sacrocolpopexy.

## METHODS

The study included patients in a large training and research hospital located in the Aegean region. Following the approval of the ethics committee and obtaining informed consent of the patients, 86 patients who underwent abdominal sacrocolpopexy between January 2014 and January 2016 which was performed by the surgical team experienced in POP were retrospectively reviewed using the hospital information management system. Readmission of the patients due to gastrointestinal complications up to two years after surgery was examined. Our clinical approach for all patients who are scheduled for prolapse surgery involves admission of the patient to the hospital one day prior to the surgery and performing bowel preparation with oral sennoside and rectal sodium dihydrogen phosphate/disodium hydrogen phosphate enema after the completion of preanesthetic interview and all other necessary consultations. The analysis included patients who were operated under epidural and spinal anesthesia in dorsal lithotomy position with Pfannenstiel incision using the same clinical approach. The patients included in the analysis were selected from the group who underwent hysterectomy and simultaneous sacrocolpopexy due to benign gynecologic reasons and symptomatic POP. All hysterectomies were performed with the modified Richardson method. Sacrocolpopexy was completed by fixing the prolene mesh (Polymesh, Betatech^®^, Istanbul, Turkey) between the anterior longitudinal ligament exposed by opening the peritoneum over the sacrum and vaginal cuff with prolene suture (Surgipro®, Covidien, CT, USA) and peritonealizing over the mesh after achieving hemostasis. Patients whose data of pre-, peri-, or postoperative opioid use and time to postoperative gas-stool passage were available in the hospital information system were included in the study. Physical examination findings on admission, age, body mass index, previous history of abdominal surgery, operation time, estimated blood loss, surgical methods, intraoperative complications and operation times, postoperative daily examination findings, and patient discharge information in the hospital information system were reviewed.

The patients were divided into two groups according to the length of hospital stay and evaluated in terms of gastrointestinal complications. Our usual clinical approach involves discharging the patients who do not have postoperative nausea, vomiting, abdominal distension, or subileus–ileus on postoperative day two and asking the patients to come for follow-up visits at postoperative one and six months; accordingly, patients with a hospitalization time of >2 days were considered to have a prolonged length of hospital stay. The data of patients from the hospital information system were reviewed for imaging studies, consultations, therapies and dietary changes related to their gastrointestinal symptoms, nausea, vomiting, and other complaints.

### Statistical Analysis

The SPSS version 22.0 software program was used for statistical analysis. An independent t-test was used for continuous parametric variables, the Mann–Whitney U test for nonparametric variables, and chi-square test for categorical variables. A p-value of <0.05 was considered statistically significant.

## RESULTS

We retrospectively reviewed 86 patients who underwent surgery for symptomatic POP by a single experienced surgeon in our hospital between January 2014 and January 2016. Twenty-one patients with missing data in the hospital information system and those who were lost to follow-up were excluded; finally, the study included 65 patients. These patients were divided into two groups: those with and without prolonged length of hospital stay. The demographic data are summarized in Table-I.

**Table-I:** Patient characteristics.

	Patients without prolonged length of hospital stay (n = 38)	Patients with prolonged length of hospital stay (n,%) (n = 27)	p-value
Age (years)^a^	53.61 ± 8.897	56.7 ± 11.332	0.245
Parity^b^	3.1	3.6	0.414
Body mass index (kg/m^2^)^a^	25.57 ± 3.414	24.29 ± 3.501	0.211
Menopause^c^	18 (48%)	22 (82%)	0.367
Smoking^c^	3	2	0.676
Hypertension^c^	14	10	0.551
Diabetes mellitus^c^	4	5	0.266
History of abdominal surgery^c^	5 (13%)	8 (30%)	0.048

a Student’s t-test (mean ± standard deviation),

b Mann–Whitney U test,

c Chi-square test

The types of surgery performed on the patients are summarized in Table-II. Three patients who did not have stress urinary incontinence at the time of surgery and who did not undergo Burch colposuspension procedure underwent transobturator tape procedure at 8, 10, and 16 months after initial surgery due to occult stress urinary incontinence (OSUI). Six patients underwent posterior and anterior wall repair in the postoperative follow-up period (Table-II).

**Table-II:** Operation characteristics.

	Patients without prolonged length of hospital stay (n, %) (n = 38)	Patients with prolonged length of hospital stay (n, %) (n = 27)
*Operation type*		
TAH/BSO+ASC	17	11
TAH+ASC	6	3
TAH/BSO+ASC+BC	15	13
Operation time (min)	101.7	108.7
Pre–postoperative Hb difference	1.88	1.87
Postoperative hospitalization time (days)	1.5	4.03
Mesh erosion in follow-up (24 months)	0/38	0/27
*Reoperation for other reasons*		
Anterior colporrhaphy	2 (at 6 and 14 months) 1 (at 12 months) 1 (at 8 months)	3 (at 10, 12, and 16 months) 02 (at 8 and 16 months)
Posterior colporrhaphy		
Transobturator tape		
*POP-Q stage*		
II	9	7
III	24	18
IV	5	2

TAH: Total abdominal hysterectomy, BSO: Bilateral salpingo-oophorectomy,

ASC: Abdominal sacrocolpopexy, BC: Burch colposuspension;

POP-Q: Pelvic Organ Prolapse Quantification System.

As patients without postoperative gastrointestinal problems who can tolerate oral intake and who do not have any additional complications were discharged on postoperative day two according to the usual clinical approach, 27 out of 65 patients with a hospital stay of >2 days were analyzed for gastrointestinal symptoms. In 24 of these 27 patients, nausea and vomiting following oral intake that began on postoperative day one recovered with antiemetic drugs, hydration, and restriction of oral intake for 12 hour. Patients tolerating oral intake were discharged latest by postoperative day four, and no additional imaging studies were required. Three patients with a hospital stay of >4 days postoperatively underwent direct abdominal radiography, followed by computed tomography (CT) of the abdomen. Direct X-ray imaging showed air-fluid level in one patient and normal findings in the other two. CT showed free fluid between the bowel loops and findings not ruling out ileus. Therefore, a general surgeon was consulted. Nasogastric decompression was performed in all patients, and oral intake was stopped. Resolution occurred in all the patients latest by postoperative day six. Additionally, none of the patients had perioperative intestinal injury or postoperative hepatitis, jaundice, or gastrointestinal bleeding. Patients who tolerated oral intake and did not require additional treatment were discharged on the first day after resolution. None of the patients were readmitted, operated, or hospitalized due to gastrointestinal complications after discharge within the first 2 postoperative years (Table-III).

**Table-III:** Data of patients with prolonged length of hospital stay (or data of patients who were prediagnosed with ileus, those who did not respond to standard treatment, and those whose hospitalization time was >4 days).

Case	Postoperative hospitalization time (days)	Performed operation	Diagnosis and treatment
1	5	TAH/BSO SC	Ileus? Stopping oral intake, parenteral nutrition, nasogastric decompression
2	6	TAH/BSO SC	Ileus? Stopping oral intake, parenteral nutrition, nasogastric decompression
3	6	TAH/USO SC BC	Ileus? Stopping oral intake, parenteral nutrition, nasogastric decompression

TAH: Total abdominal hysterectomy, BSO: Bilateral salpingo-oophorectomy, SC: Sacrocolpopexy, BC: Burch Colposuspension

## DISCUSSION

The objective of our study was to evaluate the gastrointestinal complications which prolong the length of hospital stay, require readmission or repeat surgery after discharge, and increase the morbidity and mortality in patients undergoing abdominal sacrocolpopexy.

Particularly in the high-risk patient population, complaints such as nausea and vomiting following abdominal surgery occur in up to 70% of patients. Female patients, those with a previous history of postoperative nausea and vomiting, nonsmokers, and those using postoperative opioids were at the risk of experiencing postoperative nausea and vomiting. Antiemetic drugs must be used for prophylaxis in patients who are at the risk of experiencing postoperative nausea and vomiting.^13^ It has been reported that systemic opioid use is reduced and the incidence of postoperative ileus decreases with the use of epidural anesthesia.^14^ The rate of accompanying gastrointestinal complications in our patient group was 4.6%. The relatively lower rates of gastrointestinal complications in our patients than those reported in similar studies in the literature despite increased inflammation and decreased mobilization due to simultaneous hysterectomy and other gynecologic procedures may be attributed to the following factors: all our patients underwent epidural anesthesia; all the patients received perioperative antiemetic drugs; and the patients were supported with early mobilization and early oral intake. A study comparing postoperative early (six hour) and delayed (72 hour) oral intakes reported no difference in the occurrence of postoperative ileus and gastrointestinal complications^15^; however, some studies have shown that early postoperative oral intake promotes a decrease in postoperative ileus time by stimulating coordinated peristaltic activity and increasing the secretion of hormones that positively affect bowel motility.^16^

Previous studies have suggested that etiological factors for postoperative ileus include surgical trauma, general anesthesia, postoperative opioid use and inflammation.^11^ SBO was not detected in any patient during the 27-month follow-up period (range, 16–36 months), but postoperative ileus occurred in three (4.6%) patients, who were then conservatively monitored (Table-III). In a review of similar studies conducted by Nygaard et al., the incidence of postoperative ileus was reported to be 1.1%–9.3%.^8^ Perioperative bowel injury did not occur in any of our patients, whereas it occurred in 1.6% of patients reviewed by Nygaard et al.^8^ Furthermore, the incidence of gastrointestinal injury was reported to be 1.3% in a study that particularly included patients who underwent laparoscopic sacrocolpopexy.^14^ The anatomic and functional success of laparoscopic sacrocolpopexy was similar to that of the open technique .^17^,^18^

History of abdominal surgery was the only factor that significantly differed between patients with prolonged length of hospital stay due to gastrointestinal reasons and those without prolonged length of hospital stay (p < 0.05). These results were consistent with those reported in the literature.^19^,^20^ In the present study, there was no significant relationship between other variables and the length of hospital stay. There were no gastrointestinal complications requiring repeat surgery during the 27-month follow-up period. However, in a previous study, SOB was reported in one patient 14 years after sacrocolpopexy^21^; hence, the authors suggested that patients undergoing sacrocolpopexy should undergo lifelong follow-up for gastrointestinal complications. Three patients who did not undergo Burch colposuspension underwent transobturator tape procedure due to OSUI during the follow-up; the results were similar to those of our previous sacrocolpopexy analyses.^22^

### Limitations of the study

It included the retrospective study design and average follow-up time of approximately 27 months. However, strict patient selection criteria were followed, and those with missing data in the hospital information system were excluded from the analysis to mitigate these limitations. The strengths of the study included all operations performed by a single surgical team, postoperative follow-ups conducted by the same team, and same material used for all patients for sacrocolpopexy. Prospective, randomized, multicenter follow-up studies on abdominal sacrocolpopexy may further contribute to the existing literature.

### Author’s Contribution

**AHI:** Conceived, designed and did statistical analysis & editing of manuscript.

**EB:** Did data collection and manuscript writing.

**AGK & AB:** Did review and final approval of manuscript.
